# Racial and ethnic disparities in access to community-based perinatal mental health programs: results from a cross-sectional survey

**DOI:** 10.1186/s12889-024-18517-7

**Published:** 2024-04-20

**Authors:** Slawa Rokicki, Mitu Patel, Patricia D. Suplee, Robyn D’Oria

**Affiliations:** 1 Department of Health Behavior, Society, and Policy, Rutgers School of Public Health, Piscataway, NJ USA; 2 Geary Institute for Public Policy, University College Dublin, Dublin, Ireland; 3 School of Nursing-Camden, Rutgers University, Camden, NJ USA; 4 Central Jersey Family Health Consortium, North Brunswick Township, NJ USA

**Keywords:** Perinatal depression, Maternal health, Peer support groups, Racial disparities, Access to care, Community-based interventions

## Abstract

**Background:**

Perinatal mental health is a major public health problem that disproportionately affects people from racial and ethnic minority groups. Community-based perinatal mental health programs, such as peer support groups, are essential tools for the prevention and treatment of perinatal depression. Yet, little is known about racial and ethnic disparities in accessibility and utilization of community-based perinatal mental health programs.

**Methods:**

We conducted a cross-sectional study using an online survey with program administrators representing perinatal mental health community-based services and support programs throughout New Jersey. Descriptive analysis and mapping software was used to analyze the data.

**Results:**

Thirty-three program administrators completed the survey. Results showed substantial racial and ethnic disparities in availability and utilization of community-based programs. In the majority of programs, Black, Hispanic, and Asian individuals made up less than 10% of total annual participants and less than 10% of facilitators. There were also geographic disparities in program accessibility and language availability across counties. Program administrators identified mental health stigma, lack of support from family, fear of disclosure of mental health challenges, social determinants, lack of language-concordant options in programs, and limited awareness of programs in the community as significant barriers to participation of racial and ethnic minorities. Strategies to address barriers included adding language options, improving program outreach, and increasing diversity of facilitators.

**Conclusions:**

This study provides new evidence on racial and ethnic disparities in access to community-based perinatal mental health programs. Efforts to build the resources and capacities of community-based programs to identify equity gaps, increase diversity of staff, and address barriers to participation is critical to reducing racial and ethnic inequities in perinatal mental health.

**Supplementary Information:**

The online version contains supplementary material available at 10.1186/s12889-024-18517-7.

## Background

Perinatal depression (PD) is a major public health problem that disproportionately affects racial and ethnic minority groups [1, 2]. Recent data indicates that mental health conditions are the leading cause of pregnancy-related deaths among Hispanic women [3]. Individuals from lower-income and immigrant populations also have high rates of PD [4]. Structural racism shapes inequities in the prevalence, diagnosis, and treatment of perinatal mental health conditions [5, 6]. Black and Hispanic people are less likely to be screened for PD than their White counterparts [5, 7], and are less likely to seek treatment when they experience symptoms [8]. Structural barriers to accessing mental health treatment for racial and ethnic minority groups include lack of insurance coverage, limited availability of mental health professionals, and social determinants like lack of transportation or childcare [9–11]. In addition, a legacy of prejudice and discrimination in health care have resulted indistrust of health care providers, fear of involvement of child protection services, fear of discrimination,–, and internalized stigma of mental illness as substantial barriers to seeking professional mental health care [9–13, 34].

Social support is a significant protective factor for PD [14–16]. Both emotional support (love and encouragement from partner, family, and peers) and instrumental support (material goods and services like housekeeping help) are associated with lower risk of PD among postpartum people [16–19]. Qualitative research finds that support from family, friends, and peers is a particularly beneficial factor for people from racial and ethnic minority groups experiencing PD [9].

Community-based PD programs, such as peer support groups, are essential tools for the prevention and treatment of PD. In peer support groups, individuals who have faced similar experiences come together to share their views, discuss and validate each other’s experiences, and exchange helpful resources, often with the aid of a professional facilitator [20]. A recent meta-analysis found that peer support groups for perinatal women reduce feelings of isolation and inferiority, buffer the harmful effects caused by stressors, improve maternal self-efficacy, and are effective in preventing and treating depression [21]. Peer support can also increase adherence to mental health treatment such as antidepressants or counseling [22]. Moreover, peer support is often a preferred treatment pathway for PD among racial and ethnic minorities over professional mental health services due to concerns about taking antidepressants during pregnancy or while breastfeeding, negative experiences or poor rapport with mental health professionals, and difficulty in overcoming the aforementioned barriers to accessing mental health care [9, 19].

Improving access to peer support and other community-based perinatal mental health programs for racial and ethnic minorities is critical to increasing perinatal mental health equity, yet there may be significant barriers to accessing these programs. Nationally, White people and those with higher education are overrepresented among those that use online peer support groups for mental health, while racial and ethnic minority groups are underrepresented [23]. Due to cultural and language differences, individuals may feel isolated in a support group made up of participants and facilitators whose racial or ethnic identities do not match their own [19, 24]. Moreover, programs may be less available or accessible in areas where racial and ethnic minorities reside, while social determinants like lack of transportation, safety of neighborhoods, or challenges in internet access may hinder participation.

Identifying and reducing inequities in access to community-based perinatal mental health programs is an important maternal health priority, however little is known about racial and ethnic disparities in access and utilization of these programs [25]. We conducted a survey of administrators representing perinatal mental health community services and support programs available to childbearing individuals in New Jersey, a diverse state with large racial and ethnic disparities in PD. In 2019, self-reported data from a New Jersey population-level survey found that rates of PD were 17.3% among Asians, 14.3% among non-Hispanic Blacks, 10.6% among Hispanics, and 7.2% among non-Hispanic Whites [26]. The goals of our study were to: (1) examine utilization and facilitation of community-based perinatal mental health programs by race and ethnicity, (2) examine accessibility of programs by geographic location and language availability, (3) describe barriers to accessing programs by racial and ethnic minority groups, and (4) identify strategies to address these barriers.

## Methods

The study was conducted as part of an academic-community partnership between Rutgers School of Public Health and Central Jersey Family Health Consortium of New Jersey (CJFHC). CJFHC administrators provided support on the design and piloting of the survey instrument, outreach and recruitment of survey participants, and interpretation of survey results.

We first conducted an audit to identify community-based perinatal mental health programs in New Jersey, drawing on CJFHC’s local knowledge and experience as well as a variety of Internet-based resources (e.g., Perinatal Support International, NJ211.org, NJ Department of Health). After the initial survey was drafted, a pilot study was conducted with two program administrators at CJFHC. After collecting their feedback and comments, revisions to the survey questions were completed. The final survey included 15 items, composed of program characteristics, demographic composition of program participants and facilitators, and barriers and strategies to increasing access for participants from racial and ethnic minority groups and non-English speaking participants. The complete list of survey questions and response options can be found in Appendix Table 1. The survey was implemented online through REDCap.

**Table 1:** Characteristics of community perinatal mental health programs in New Jersey (*n* = 33)

Characteristic	Percent of programs choosing option (%)
**Purpose of program**	
New mothers support group	61
Perinatal depression support group	40
Parent Education	39
One-on-one counseling	21
Urgent mental health/mental health crisis	9
Crisis pregnancy	6
Drug, alcohol, or substance support	6
Other (e.g. Doula support, referrals)^1^	42
**Target participants**	
Postpartum women	94
Pregnant women	70
Parents	52
Teens	12
Children	12
Other (e.g. partner, provider)^2^	18
**Length of participant involvement in program**	
Repeating multiple sessions	58
Drop-in sessions	42
One-time, with referral	18
One-time, no referrals	15
Other^3^	27
**Type of Facilitator**	
Mental health professional	58
Other professional (e.g. RN, APN, MSW)^4^	61
Peer	30
Other^5^	12
**Format of program**	
Virtual	82
Phone	42
In-Person	42
Text	24
Other (e.g. Facebook, Email)^6^	9
**Language options available**	
English	91
Spanish	36
Chinese (Mandarin)	6
Portuguese	12
Interpreter services	18
Other (e.g. Creole, French)^7^	3
**Cost of program**	
Free	70
One-time fee	3
Recurring fee	9
Other (e.g. Various fees, sliding scale, insurance)^8^	18
**Type of program**	
Non-profit based	61
Hospital based	39

*Notes*: Survey questions allowed for multiple responses so percentages do not add up to 100%. ^1^For the purposes(s) of program, other responses included: Emotional Health Phone Support Program, Referrals to lactation or mental health professionals, Web-based screening and psychiatric evaluation, Doula support, Diaper distribution, Perinatal loss support, and Social peer support. ^2^For the target participants, other responses included: Partner/Fathers, Providers, and Babies. ^3^For the length of participant involvement in program, other responses included: Weekly or biweekly, Consecutive programs (for a few weeks or months), 0–12 months after birth, Specific number of follow-up calls, and Treatment as needed. ^4^For the type of facilitator, other professionals included: Registered Nurse (RN), Advanced Practice Nurse (APN), Nurse practitioner, PD Nurse, Childbirth and/or Lactation consultant, Perinatal Mental Health Certified (PMH-C) personnel, Doctors, Social worker (MSW), Community partner, and Doula. ^5^For the type of facilitator, other responses included: Health educators and Volunteers/staff to prepare orders of resources such as diapers, baby food, etc. ^6^For the format of the program, other responses included: Facebook and Email. ^7^For language options available, other responses included: Creole or French. ^8^For the cost of the program, other responses included: Some services free while some fee-for-service, sliding scale, and fee but insurance coverage accepted.

All organizations in NJ with identified perinatal mental health programs were invited to participate in the survey, conducted between November 30th, 2021 and July 1st, 2022. Participation was voluntary. Program administrators were contacted a minimum of two times via emails, phone calls, text messaging, and filling out website contact forms. Those who responded were provided with a link to the survey. CJFHC also facilitated in recruiting program administrators with whom they work in the community via email. All procedures of this research study were approved by the institutional review board at Rutgers School of Public Health.

A descriptive analysis was carried out to summarize program characteristics. To examine utilization and facilitation of programs, we calculated for each racial and ethnic group the percentage of programs who reported that their participants (or facilitators) composed less than 10% of the total annual participants (or total facilitators), which we defined as “very low participation”. ArcMap Desktop was used to create a choropleth map of the number of programs that service each county of NJ overlaid with language option availability from collected survey data. We compared Spanish-language availability to county birth rates across race and ethnicity from 2020 to 2021 using NJ state-level data [26]. Stata MP17 and Excel were used to analyze the collected survey data.

## Results

**Fig. 1:**
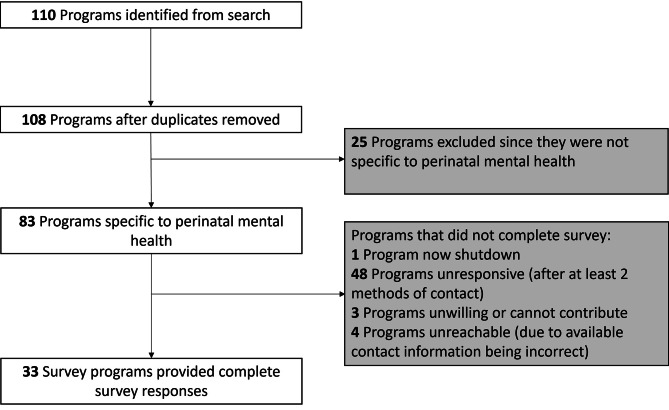
Study flow diagram

A total of 110 community-based perinatal mental health programs were identified and after removing duplicates, 108 programs remained (Fig. 1). From those 108 programs, 25 programs were excluded because they were not specific to perinatal mental health. A total of 83 perinatal mental health programs were contacted, after which one program was found to be shutdown, 48 programs were unresponsive (after at least two methods of contact), three were unwilling or unable to contribute to the survey because they did not collect the information we were asking for or because they were just launching the program, and four programs were unreachable due to available contact information being incorrect. Therefore, a total of 33 programs participated in the study.

Table 1 shows the characteristics of the programs. Among programs, 61% were characterized as new mother support groups, 40% as perinatal depression support groups, and 39% as parent education groups (program administrators could choose multiple options). The target participants for most programs were postpartum women (94%) and pregnant women (70%), while 52% also targeted parents. Most programs (58%) were repeated multiple sessions or drop-in sessions (42%). Most programs were facilitated by either a mental health professional (58%) or other health professional (61%), such as Registered Nurses, Advanced Practice Nurses, social workers, and lactation consultants. In terms of format, 82% of programs provided a virtual option, 42% of programs provided a phone option, and 42% provided an in-person option. Among programs, 36% offered services in Spanish, while 18% of programs reported that interpreter services were available. About 70% of programs were available free of cost to participants, while 12% either had a one-time fee or a recurring fee that ranged from $15-$35. The remaining 18% of programs had fees that varied depending on the service, had sliding scales, or accepted insurance including Medicaid and Medicare. Programs were either hospital-based (39%) or non-profit based (61%).Administrators reported that the COVID-19 pandemic had both positive and negative impacts on their programs: 58% of programs reported that they changed to a virtual format, while 18% reported being shut-down for some period of time; two of the programs were actually created in response to the pandemic (Appendix Table 2).

**Table 2:** Percentage of programs reporting very low participation of facilitators and participants across racial and ethnic groups

	Percentage of programs reporting very low participation
**Panel A. Participants**	
Non-Hispanic White	15
Non-Hispanic Black	55
Non-Hispanic Asian	76
Hispanic	58
Other race	82
Non-English speaking	73
**Panel B. Facilitator**s	
Non-Hispanic White	18
Non-Hispanic Black	82
Non-Hispanic Asian	97
Hispanic	61

*Notes*: Very low participation is defined as less than 10% of total annual participants and total facilitators in the program

**Fig. 2:**
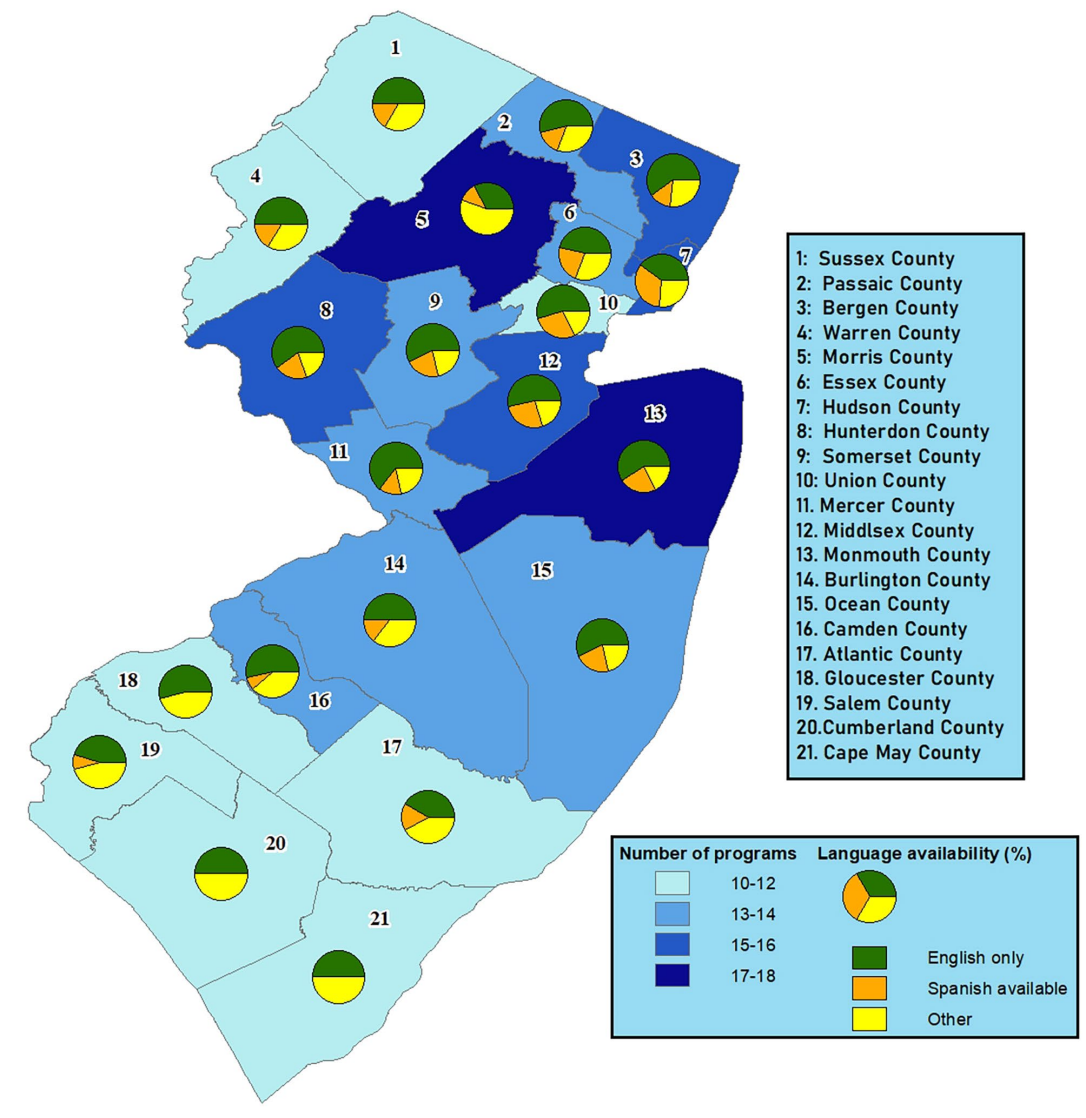
Geographic distribution of community-based perinatal mental health programs across counties of New Jersey, overlaid with availability of Spanish-language programs *Notes*: ‘Spanish available’ refers to programs that offer a Spanish-language option. ‘Other’ includes programs that do not offer Spanish directly but may offer interpretive services or other language options

Figure 2 shows a county-level map depicting the number of perinatal mental health programs that provide services in each county, overlaid with pie charts showing availability of Spanish-language and other language programs. Spanish was selected as the main language of interest as the Hispanic population is the second largest in NJ after non-Hispanic White [26]. Results show geographic disparities in the number of available programs across counties, with Northern and central counties offering more programs compared to southern counties. There were also disparities in availability of languages offered across counties. In northern counties such as Essex, Union, and Hudson, up to 33% of the programs in those counties offered programs in Spanish, while in southern counties including Gloucester, Cumberland, and Cape May, there were no programs offered in Spanish. This contrasts sharply with the population of Hispanic mothers in these southern counties: for example, 49% of the 2020–2021 births in Cumberland County were to Hispanic mothers (Appendix Table 3).

**Table 3:** Barriers and strategies to address barriers to participation of racial and ethnic minority groups in community perinatal mental health programs

	Percentage of programs responding yes
**Panel A: Barriers to participation of racial and ethnic minority groups**	
Lack of language options for non-English speakers	55
Stigma associated with mental health	39
Lack of knowledge/awareness of program in community	33
Lack of support from family	30
Lack of diversity in area serviced by program	30
Problems with internet issues	24
Transportation	21
Fear of disclosure or deportation due to disclosing mental health problems	18
Time/Schedule conflict	15
Childcare	15
Services offered are not as culturally responsive as they could be	3
Program cost	0
Other^1^	9
**Panel B: Strategies to address barriers**	
Adding language options	58
Improving program outreach and advertisement	55
Increasing diversity of facilitators	48
Increasing options for moms in certain locations	42
Conducting evaluation of services offered	24
Providing transportation	18
Providing childcare resources	18
Adding other types of program formats	18
Other (improve awareness of perinatal depression and reduce stigma; increasing provider education to improve quality of patient/client engagement)	9

*Notes*: Survey response allowed for multiple responses so percentages do not add up to 100%. ^1^Other prompts for barriers included: Other priorities, general distrust for professionals, stigma/fear of disclosing personal information about self and baby

Table 2 shows the percentage of programs reporting very low participation of program participants and facilitators by racial and ethnic groups (very low defined as less than 10% of total annual participants or total facilitators, respectively). The majority of programs reported very low participation by Black, Hispanic, and Asian individuals, that is, in the majority of programs these groups each made up less than 10% of total annual participants and less than 10% of facilitators. Additionally, 73% of programs reported very low participation by non-English speaking participants. Conversely, only 15% of programs reported very low participation by non-Hispanic White participants. Appendix Table 4 shows the full distribution of participants and facilitators across programs.

Program administrators reported on the barriers to program participation and strategies that they believed could help make perinatal mental health programs more accessible to members of racial and ethnic groups (Table 3). Reported barriers included lack of language options (55% of program administrators identified this barrier), lack of knowledge/awareness of the program in the community (33%), stigma associated with mental health (39%), lack of support from family (30%), and fear of disclosure or deportation due to disclosing mental health problems (18%). Social determinants were also reported as barriers including challenges with transportation (21%), internet access (24%), and childcare (15%).

To address these barriers, program administrators reported that adding language options (58%), improving program outreach and advertisement (55%), increasing diversity of facilitators (48%), and increasing options for participants in specific locations (42%) would be effective strategies (Table 3). Addressing social determinants like providing transportation (18%) and childcare resources (18%) were also identified as potential strategies. Three program administrators also provided additional strategy recommendations to improve awareness of perinatal depression and reduce stigma, which included promoting public campaigns that normalize perinatal mental health issues and demonstrate the racial and ethnic diversity of affected individuals. One administrator also recommended increasing provider education to improve quality of patient/client engagement.

## Discussion

In this cross-sectional survey of community-based perinatal mental health programs and support services of New Jersey, we found substantial racial and ethnic disparities in availability and utilization of programs. In the majority of programs, Black, Hispanic, and Asian individuals made up less than 10% of total annual participants. Considering the importance of social support for prevention and treatment of perinatal depression [15, 16, 20], these disparities are significant obstacles in reducing racial and ethnic disparities in perinatal mental health conditions.

Results also showed substantial geographic disparities in availability of programs, further limiting access for individuals in counties with few programs. However, as a result of the COVID-19 pandemic and the subsequent increase in perinatal mental health conditions [36], many community-based programs began to offer their programs virtually, which may have increased accessibility in geographic areas with few in-person programs. Many programs have continued to offer virtual programs, even several years after the height of the pandemic. Nevertheless, inadequate internet connectivity and lower digital literacy among racial and ethnic minority groups may prevent individuals from accessing virtual programs [27]. In our survey, a quarter of program administrators cited challenges with internet as a barrier to participation.

We also found that there were few facilitators from racial and ethnic minority groups providing services in community perinatal mental health programs. In the majority of programs, fewer than 10% of facilitators were non-Hispanic Black, Hispanic, or Asian. Moreover, few programs offered services in languages other than English. While interpreter services were available for some programs, participants may not want to share sensitive information about their mental health with an interpreter. Culturally competent care is instrumental in understanding and meeting the unique needs and experiences of individuals from racial and ethnic communities [28]. Research finds that individuals from racial and ethnic minority groups who share the same race and ethnicity with their provider have improved communication, better perceptions of care, and better health outcomes [29]. This likely extends to facilitators of peer support groups, where participants of racial and ethnic minority groups may feel isolated and misunderstood in groups without racially congruent facilitators or peers, though further work to investigate the needs and experiences of racial and ethnic minority participants regarding racial and ethnic concordance in peer support groups and other community-based programs is critical.

## Policy implications

This study contributes new evidence on racial and ethnic disparities in access to community-based perinatal mental health programs. Recent years have seen a large increase in promotion of community-based initiatives to address the mental health crisis in the United States, particularly for maternal mental health [30, 31]. For example, federal and state funding has catalyzed a significant expansion of community health workers and other paraprofessionals to provide mental health services, as a way to address the well-studied racial and ethnic barriers to accessing professional mental health services [32, 33]. However, our study provides important evidence that community-based programs have very similar access barriers to these services– both structural and individual– that racial and ethnic minority groups face. Program administrators identified mental health stigma and lack of support from family, as well as social determinants such as limited transportation and internet connectivity as substantial barriers to access, consistent with the wider literature on barriers to access to professional mental health services and treatment [9–13, 32]. Moreover, fear of deportation due to disclosure of mental health challenges was also frequently cited as an important barrier, underscoring that community-based health programs are not immune from the legacy of structural racism, discrimination, and maltreatment that has led to Black and Hispanic groups’ entrenched mistrust of the health care system [34]. These barriers restrict access of racial and ethnic groups to community-based programs and may further perpetuate racial and ethnic disparities in untreated mental health conditions among pregnant and postpartum people.

The most common strategies identified by program administrators to address access barriers were adding language options, improving program outreach and advertisement, and increasing diversity of facilitators. Operationalizing these strategies would require significant investment in the resources of community-based organizations, for example, by increasing the number of federal and state-level grants provided to grassroots organizations to support their efforts to reach racial and ethnic members of their communities who struggle with perinatal mental health issues. Such grants could aim to improve diversity of program facilitators or develop collaborative partnerships with universities or government agencies to broaden expertise and increase program reach sustainably [30]. Additionally, building the capacity of community organizations to identify equity gaps in access and adapt programs for racial and ethnic minority groups is critical [5]. For example, adaptations to promote health equity have been implemented for perinatal psychiatry access programs, though evidence on the effectiveness of these adaptations is lacking [35].

This study had several strengths. First, the use of a state-wide audit allowed us to systematically identify community-based perinatal mental health programs in NJ. Second, we examined both barriers and strategies to addressing barriers in improving access to community-based programs. Further work should consider investigating the comparative effectiveness of various strategies on reducing disparities in access to community-based programs. Third, results represent the views of the program administrators who have direct contact with populations participating in their programs. Fourth, our community partner played a pivotal role in the design and implementation of the study, which ensured high quality data collection and interpretation.

This study also has limitations. First, despite a careful audit, our study may not have captured all community-based perinatal mental health programs in NJ. Moreover, the overall survey response rate was 40%; it was not possible to ascertain if unresponsive programs were still active or not. Second, data were self-reported by program administrators and some information may not be comprehensive. Third, results may not be generalizable to other states. Fourth, this survey focuses only on program administrators and their viewpoints regarding the mental health programs they run; additional research with program participants would provide further context on the preferences of racial and ethnic minority groups regarding the types and formats of programs, and identify additional access barriers and intervention strategies.

## Conclusions

This study finds that racial and ethnic disparities in access to perinatal mental health care extends to community-based programs and services like peer support groups. Unequal access to community-based programs further perpetuates racial and ethnic disparities in treatment and prevention of perinatal mental health conditions. Efforts to build the resources and capacities of community-based mental health programs to identify equity gaps, increase diversity of staff, and address barriers to participation is critical to reducing racial and ethnic inequities in perinatal mental health.

### Electronic supplementary material

Below is the link to the electronic supplementary material.

Supplementary Material 1

## Data Availability

All data generated or analyzed during this study are included in this published article [and its supplementary information files].
