# Supplementary material for: Racial and ethnic disparities in access to community-based perinatal mental health programs: results from a cross-sectional survey
Source: BMC Public Health. 2024 Apr 20;24:1094. doi: 10.1186/s12889-024-18517-7 (PMC11031973; doi:10.1186/s12889-024-18517-7)
Supplement: Supplementary file 1 — Supplementary Material 1 [file 12889_2024_18517_MOESM1_ESM.docx]

Racial and Ethnic Disparities in Access to Community-Based Perinatal Mental Health Programs:

Results from a Cross-Sectional Survey

BMC Public Health

Slawa Rokicki, PhD, Mitu Patel, MPH, Patricia D. Suplee, PhD, RNC-OB, FAAN, Robyn D'Oria, MA, RNC

Slawa Rokicki, Department of Health Behavior, Society, and Policy; Rutgers School of Public Health, Piscataway, NJ, USA; slawa.rokicki@gmail.com

**Appendix Table 1. Community Assessment Survey**


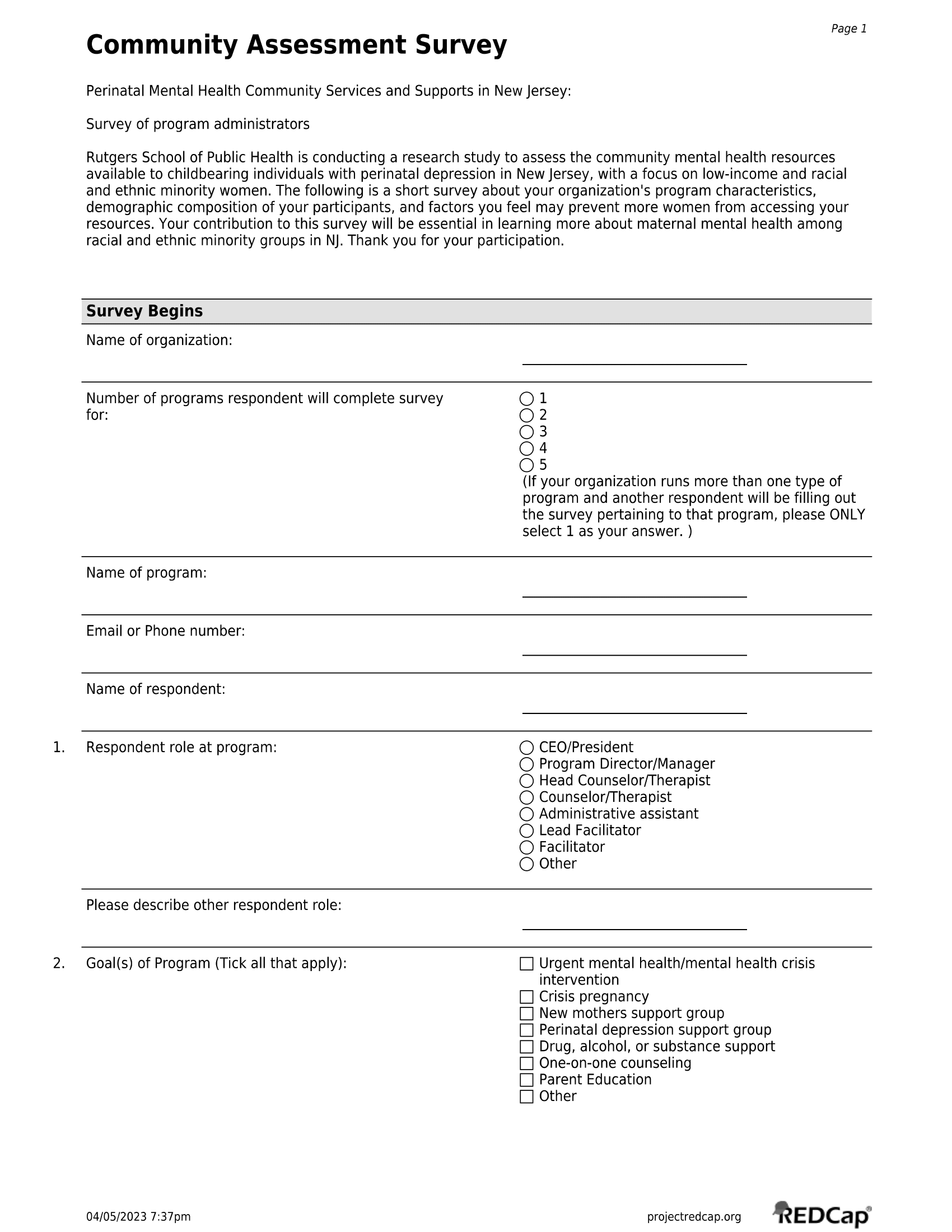


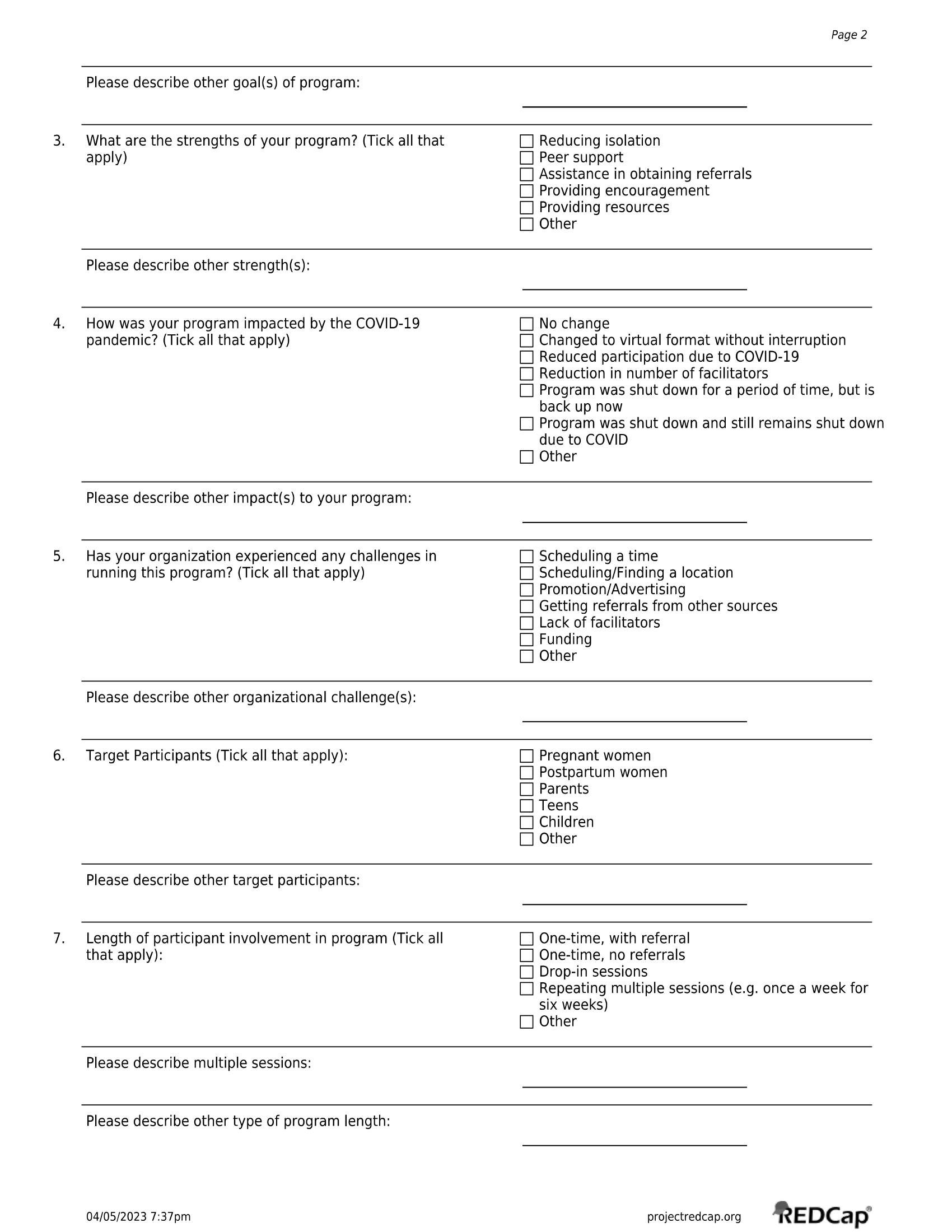


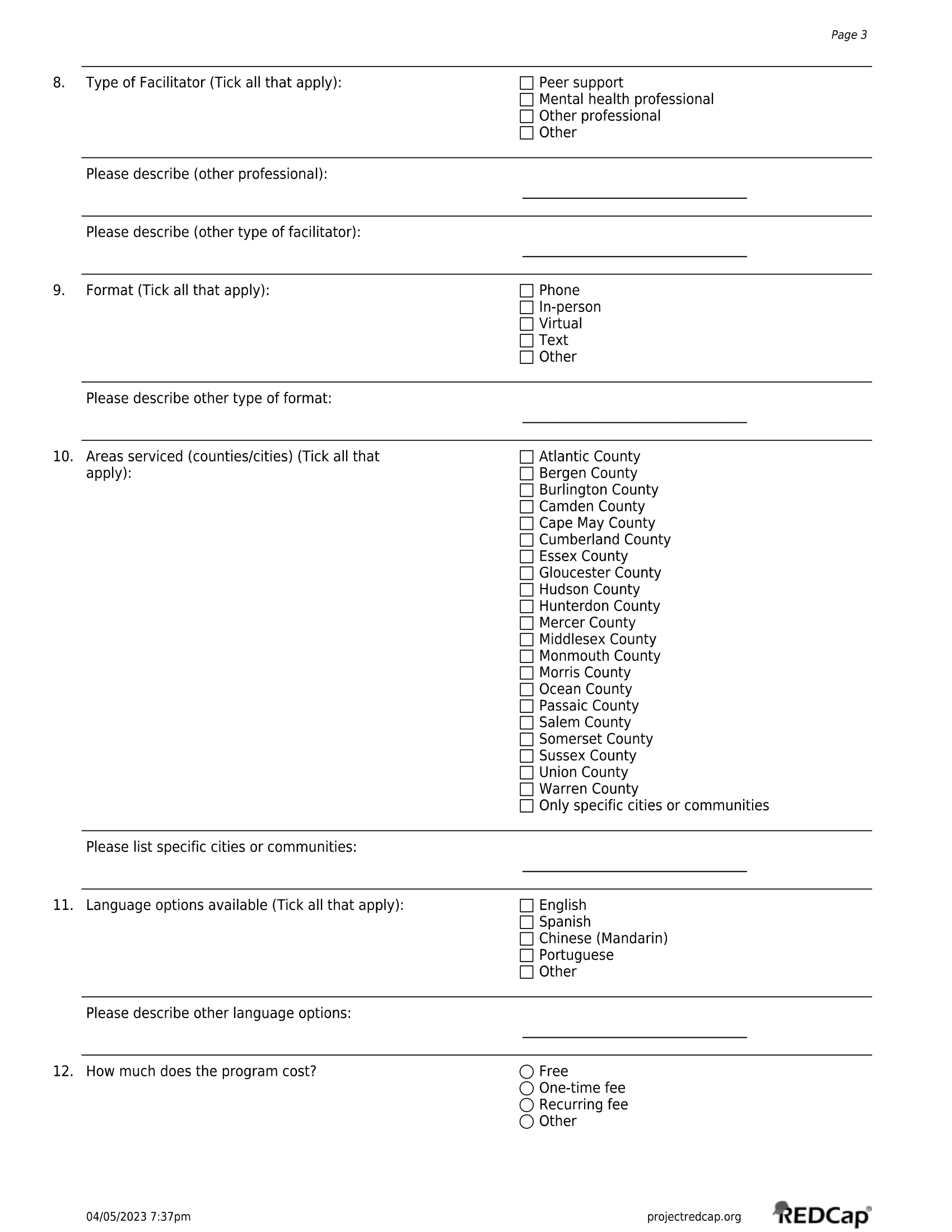


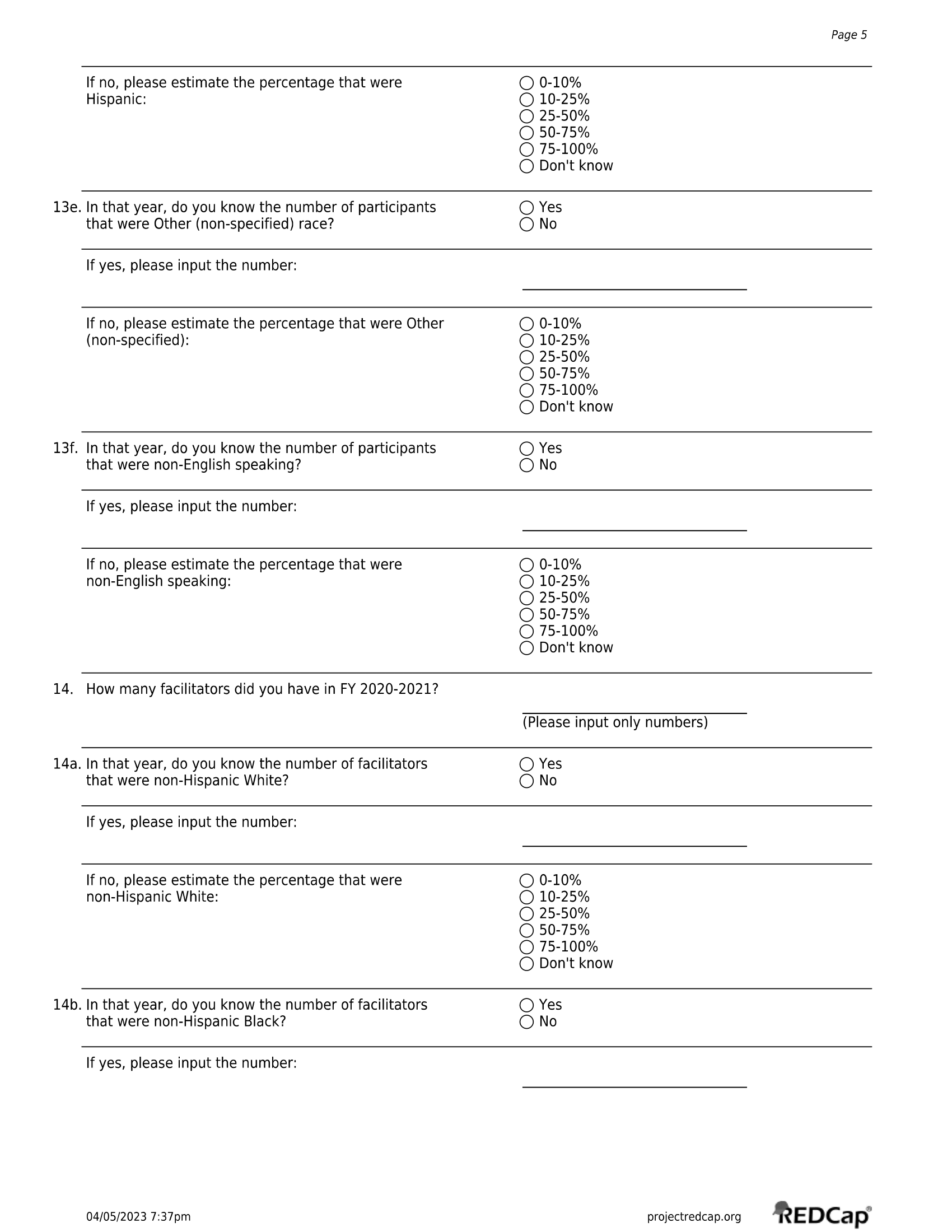


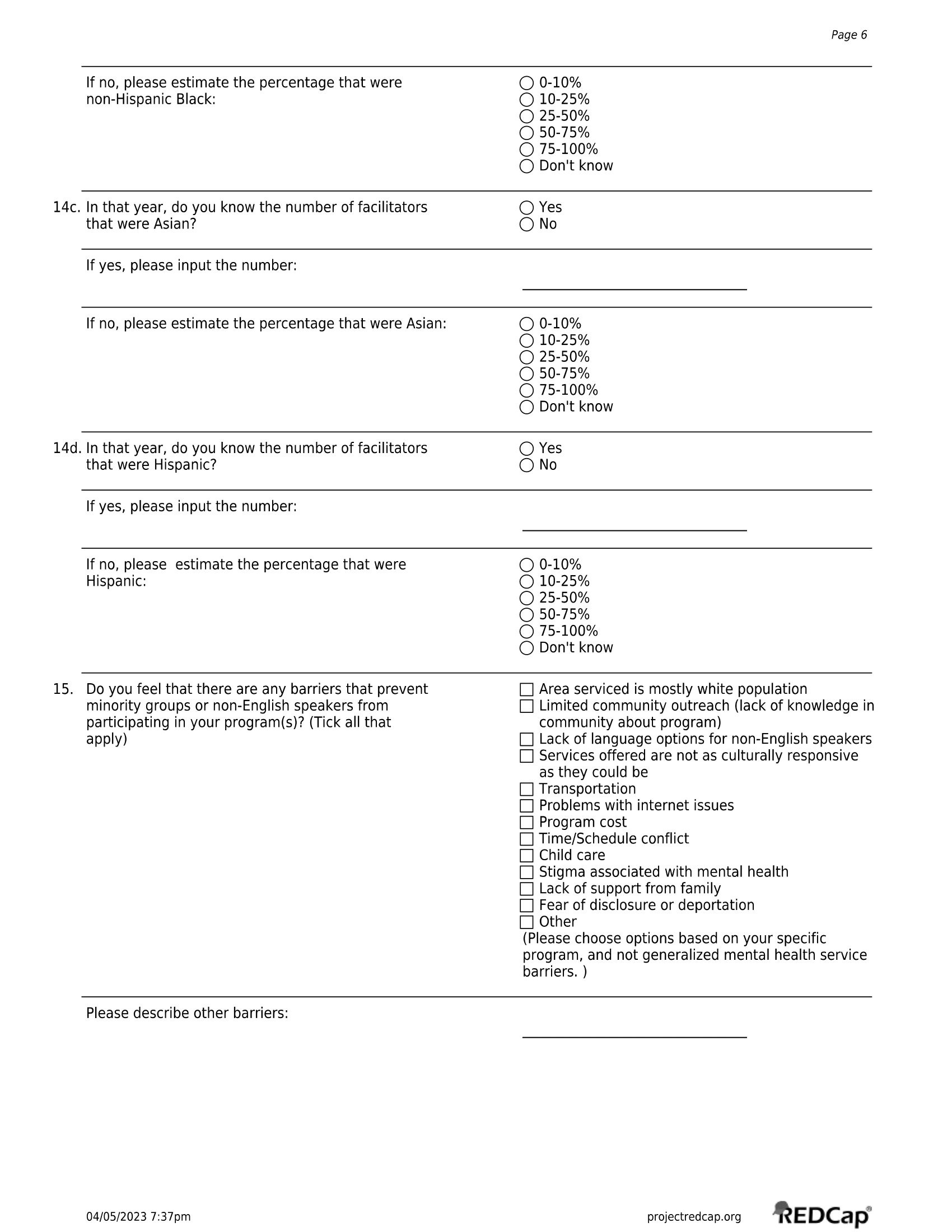


**Appendix Table 2. Survey responses for respondent role at program.**

| **Variable** | **Percent of programs** |
| --- | --- |
| **Respondent role at program** |  |
| CEO/President | 12 |
| Program Director/Manager | 30 |
| Head Counselor/Therapist | 6 |
| Counselor/Therapist | 9 |
| Administrative assistant | 3 |
| Lead Facilitator | 15 |
| Facilitator | 6 |
| Other^1^ | 18 |
|  |  |
| **Impact of COVID-19 pandemic on program** |  |
| No change | 9 |
| Changed to virtual format without interruption | 58 |
| Reduced participation due to COVID-19 | 21 |
| Reduction in number of facilitators | 0 |
| Program was shut down for a period of time, but is back up now | 15 |
| Program was shut down and still remains shut down due to COVID | 3 |
| Other^2^ | 27 |

^1^For the respondent role at program, other responses included: Perinatal Social Worker, Bilingual Family Partner, Division Director of Outpatient Services, Case Manager, Doula/Owner, Director of Development and Special Projects. ^2^Other comments for impact of COVID-19 included: added new program (2 programs), modified program in some way, increase in referrals and offering more support groups due to virtual platform, increase in participation, certain parts of programs were no longer running.

**Appendix Table 3. Availability of programs and language options by county, compared with racial and ethnic distribution of NJ births**

| **County** | **Total number of programs** | **Percent of programs with language availability (%)** | | |  | **Racial and ethnic distribution of births in NJ (%)** | | | |
| --- | --- | --- | --- | --- | --- | --- | --- | --- | --- |
|  |  | **Spanish** | **English only** | **Other** |  | **Hisp** | **NHW** | **NHB** | **NHA** |
| Atlantic | 12 | 17 | 42 | 42 |  | 30 | 45 | 18 | 7 |
| Bergen | 15 | 13 | 60 | 27 |  | 30 | 47 | 5 | 17 |
| Burlington | 14 | 14 | 50 | 36 |  | 14 | 60 | 18 | 8 |
| Camden | 13 | 8 | 54 | 38 |  | 28 | 45 | 21 | 6 |
| Cape May | 10 | 0 | 50 | 50 |  | 16 | 79 | 4 | 1 |
| Cumberland | 10 | 0 | 50 | 50 |  | 49 | 32 | 18 | 1 |
| Essex | 13 | 23 | 46 | 31 |  | 32 | 23 | 39 | 5 |
| Gloucester | 11 | 0 | 55 | 45 |  | 11 | 73 | 13 | 3 |
| Hudson | 15 | 33 | 40 | 27 |  | 43 | 24 | 11 | 22 |
| Hunterdon | 15 | 20 | 60 | 20 |  | 15 | 77 | 2 | 6 |
| Mercer | 14 | 14 | 64 | 21 |  | 38 | 31 | 21 | 10 |
| Middlesex | 15 | 27 | 53 | 20 |  | 34 | 28 | 10 | 28 |
| Monmouth | 17 | 24 | 59 | 18 |  | 20 | 70 | 7 | 4 |
| Morris | 18 | 11 | 33 | 56 |  | 21 | 62 | 3 | 14 |
| Ocean | 14 | 21 | 57 | 21 |  | 10 | 87 | 2 | 1 |
| Passaic | 13 | 15 | 54 | 31 |  | 55 | 29 | 9 | 7 |
| Salem | 11 | 9 | 45 | 45 |  | 20 | 58 | 21 | 1 |
| Somerset | 14 | 21 | 57 | 21 |  | 27 | 43 | 10 | 21 |
| Sussex | 12 | 17 | 50 | 33 |  | 13 | 81 | 2 | 3 |
| Union | 11 | 27 | 55 | 18 |  | 45 | 31 | 18 | 6 |
| Warren | 12 | 17 | 50 | 33 |  | 18 | 73 | 6 | 3 |

Notes: Data for distribution of births obtained for the years 2020-2021 from NJSHAD. Hisp=Hispanic (of any race). NHW=non-Hispanic White. NHB=non-Hispanic Black. NHA=non-Hispanic Asian. Other language availability refers to programs that may offer Chinese, Portuguese, Creole, French, and/or Interpretative services language availability

**Appendix Table 4. Distribution of participations and facilitators across community-based perinatal mental health programs (n=33)**

|  | Percent of total participants (facilitators) across programs (%) | | | | | |
| --- | --- | --- | --- | --- | --- | --- |
|  | **0-10%** | **10-25%** | **25-50%** | **50-75%** | **75-100%** | **Don’t Know** |
| Panel A. Participants |  |  |  |  |  |  |
| Non-Hispanic White | 15 | 6 | 18 | 27 | 27 | 6 |
| Non-Hispanic Black | 55 | 24 | 12 | 3 | 0 | 6 |
| Non-Hispanic Asian | 76 | 12 | 6 | 0 | 0 | 6 |
| Hispanic | 58 | 21 | 9 | 0 | 6 | 6 |
| Other race | 82 | 6 | 0 | 0 | 3 | 9 |
| Non-English speaking | 73 | 6 | 9 | 3 | 6 | 3 |
|  |  |  |  |  |  |  |
| Panel B. Facilitators |  |  |  |  |  |  |
| Non-Hispanic White | 18 | 0 | 3 | 12 | 64 | 3 |
| Non-Hispanic Black | 82 | 9 | 0 | 3 | 3 | 3 |
| Non-Hispanic Asian | 97 | 0 | 0 | 0 | 0 | 3 |
| Hispanic | 61 | 15 | 9 | 3 | 9 | 3 |
